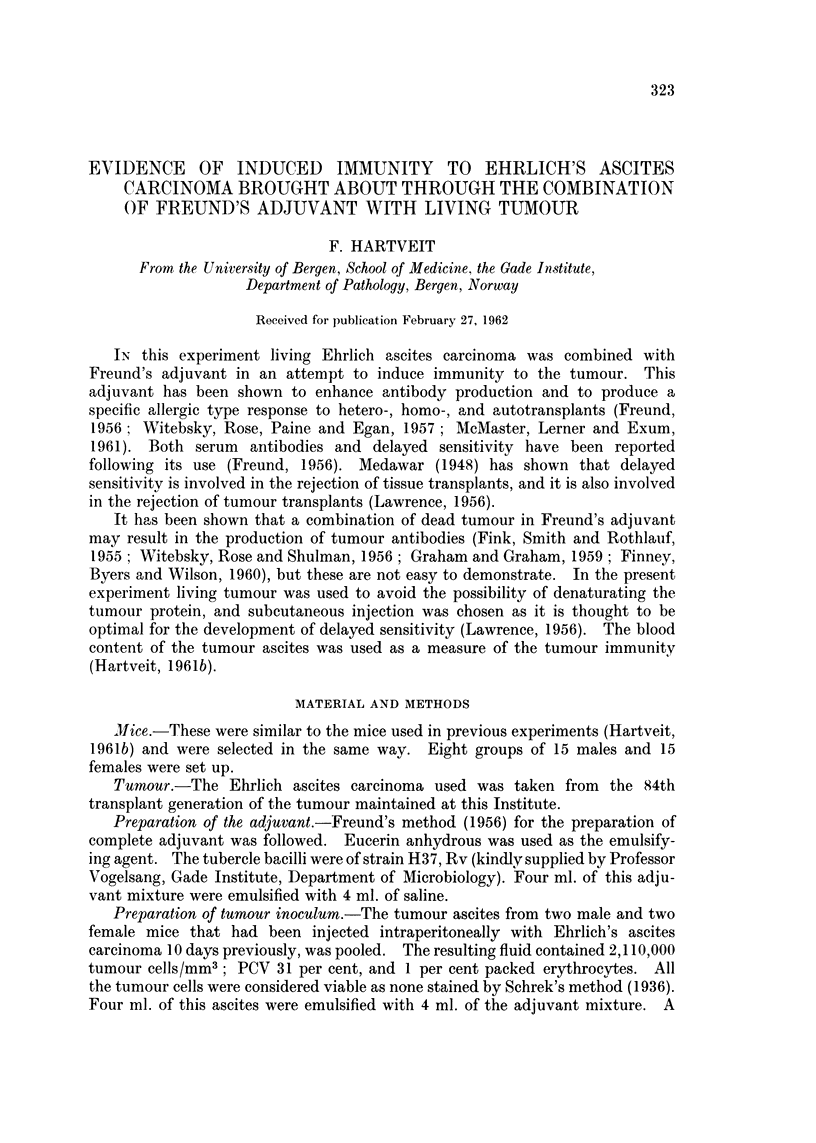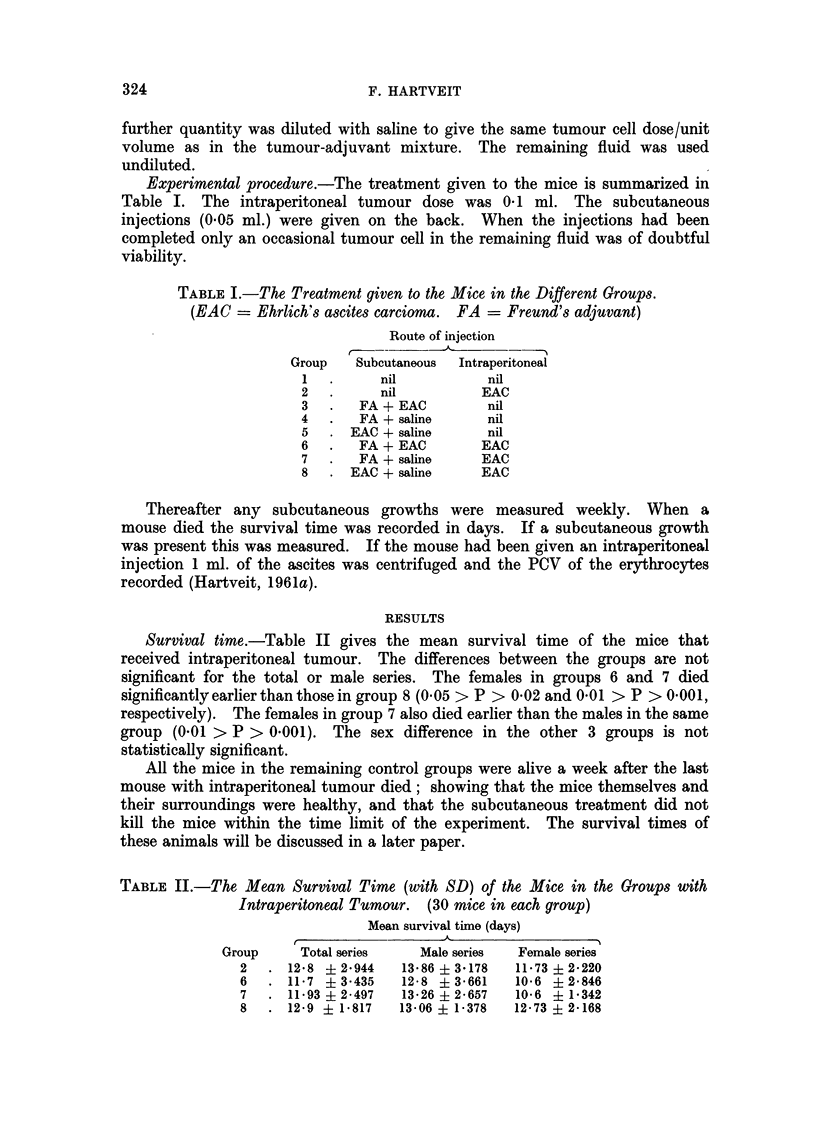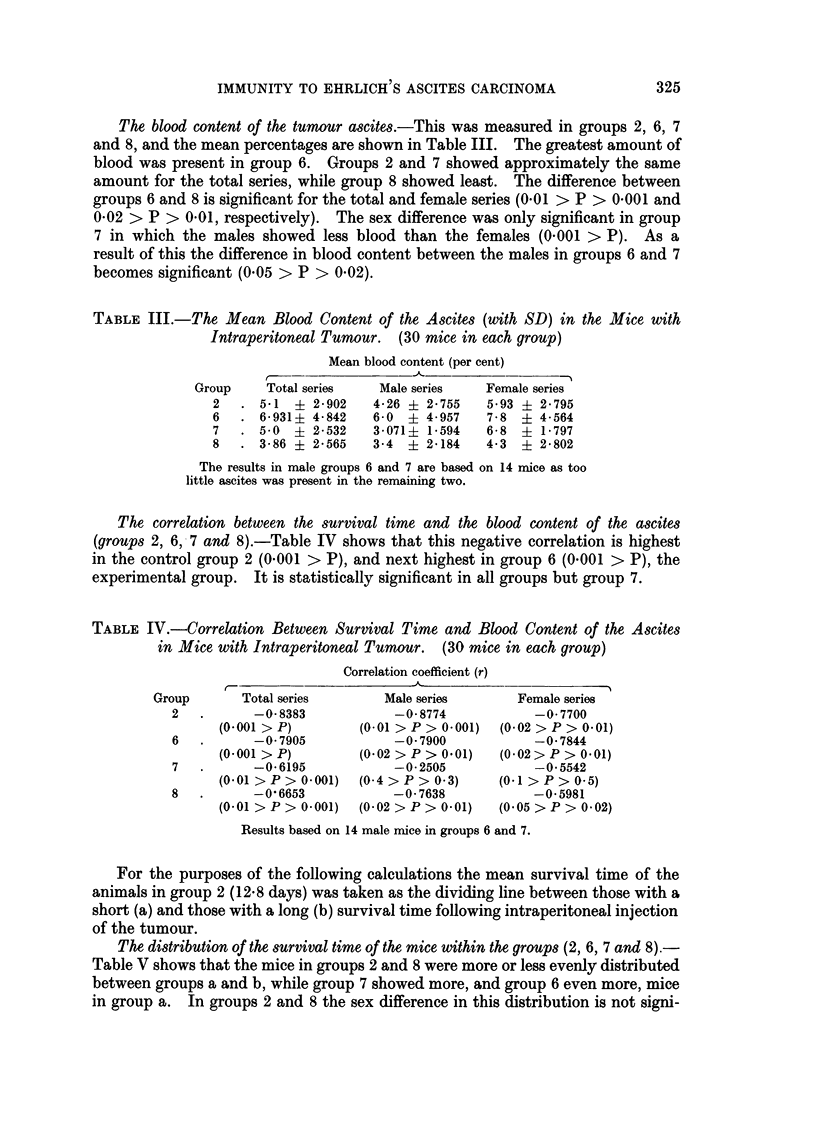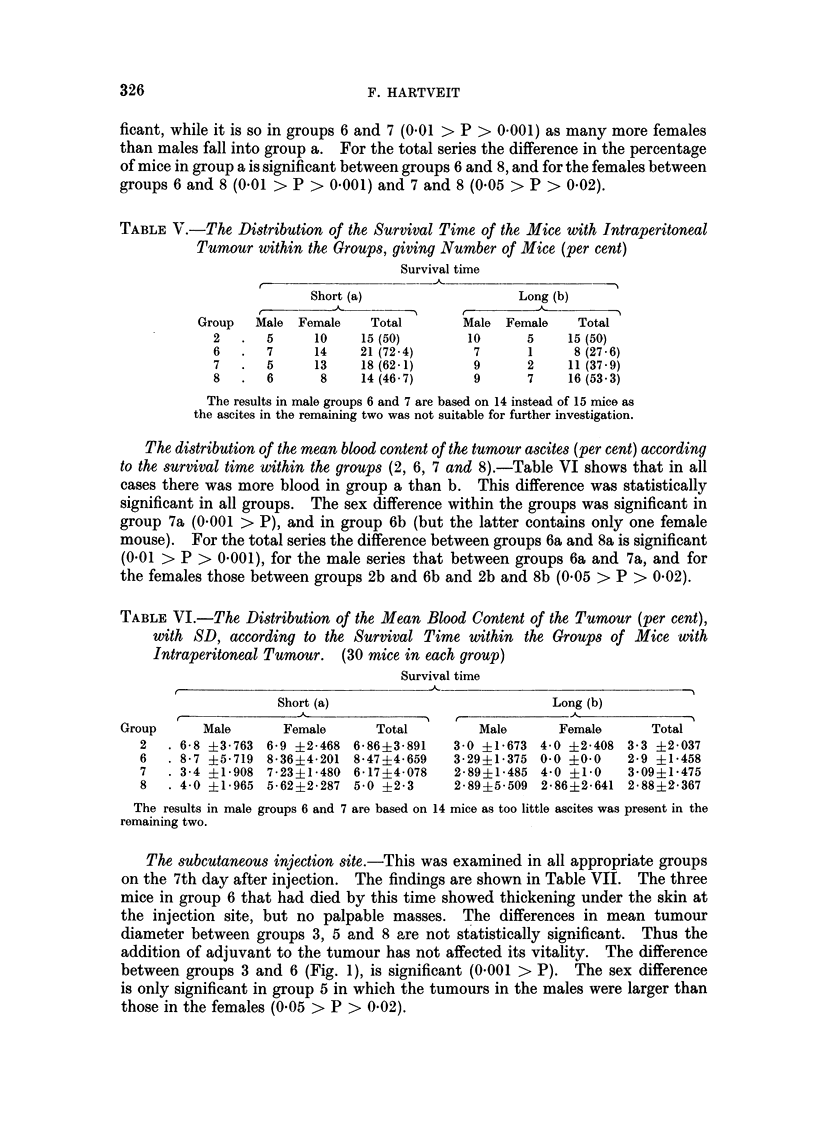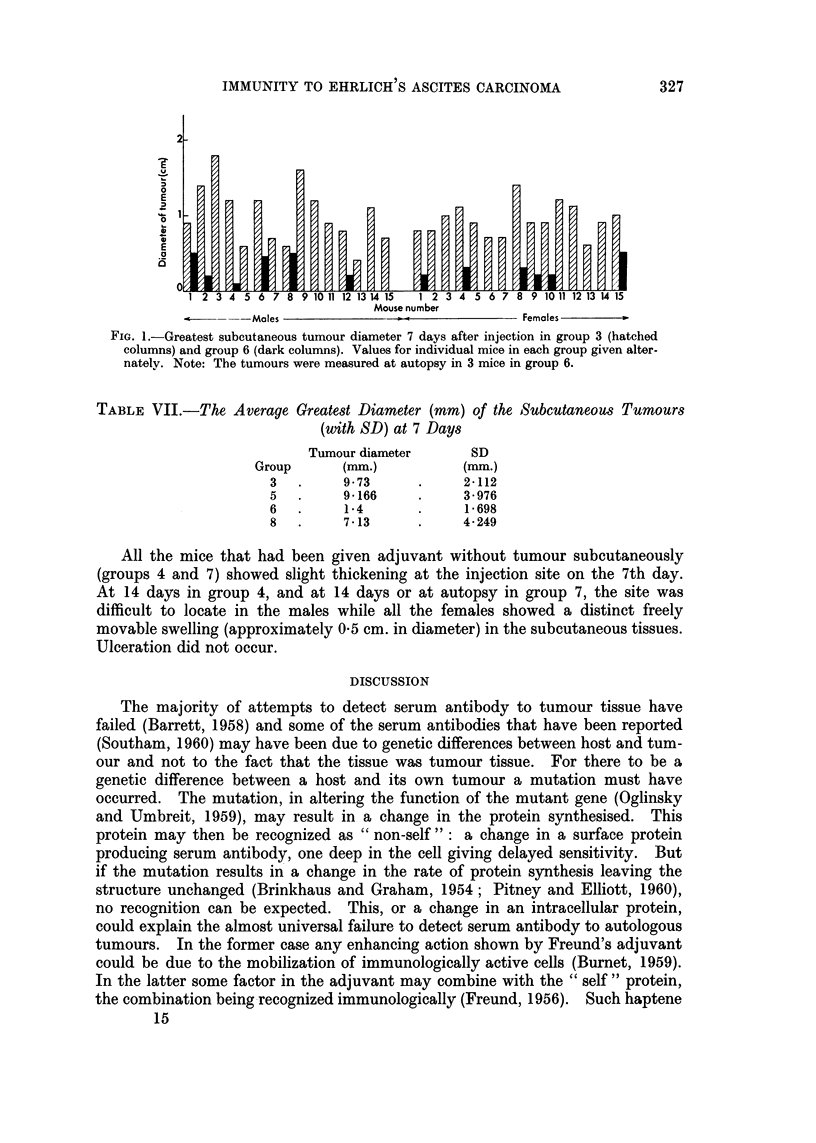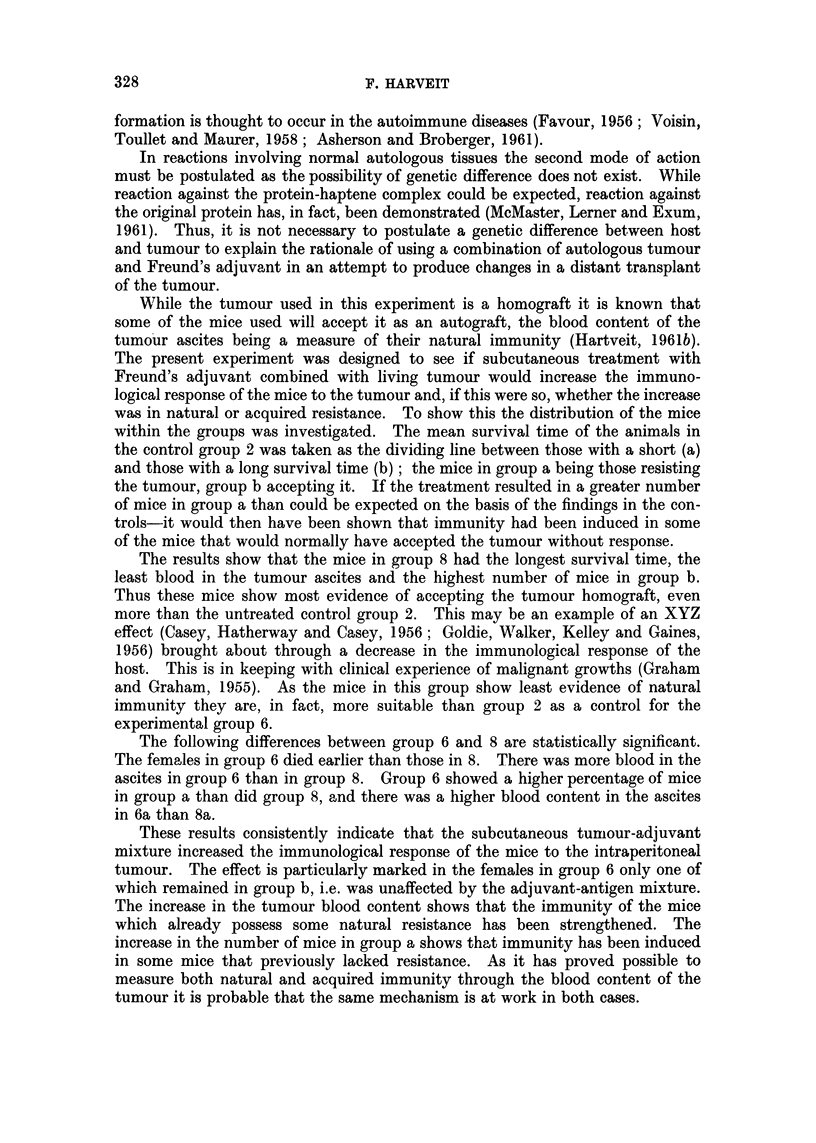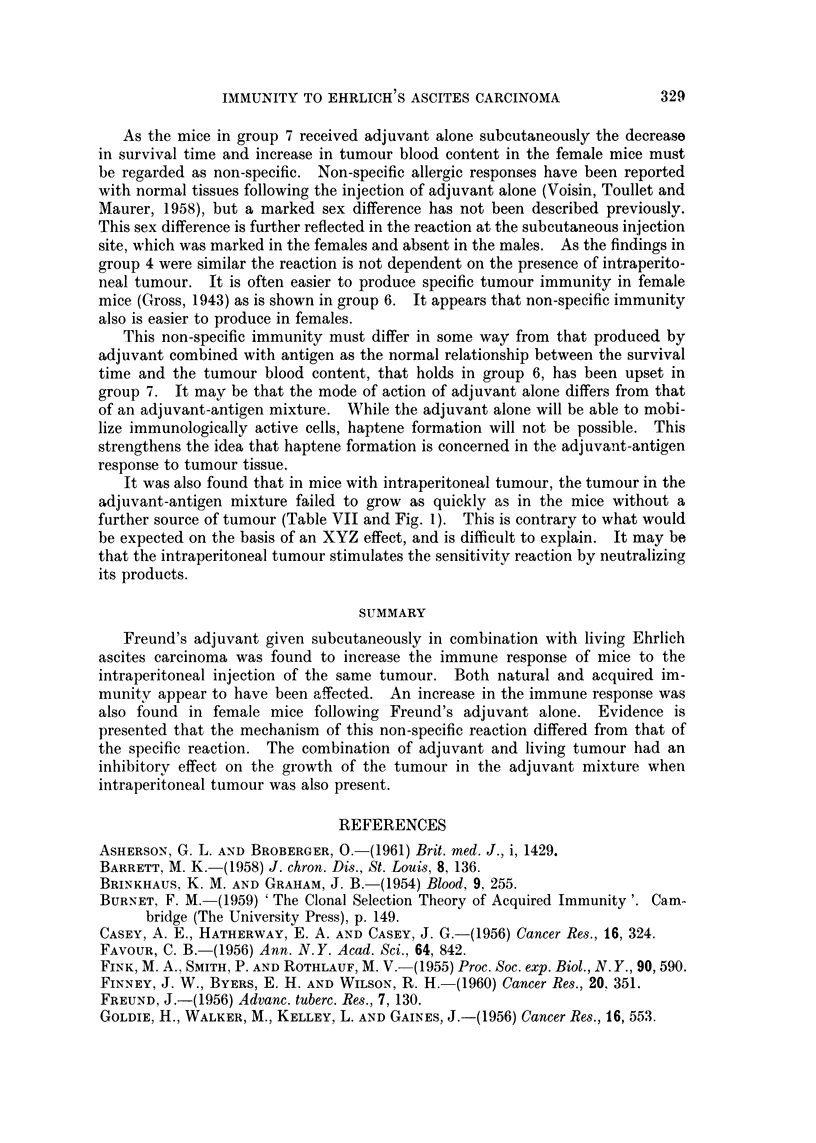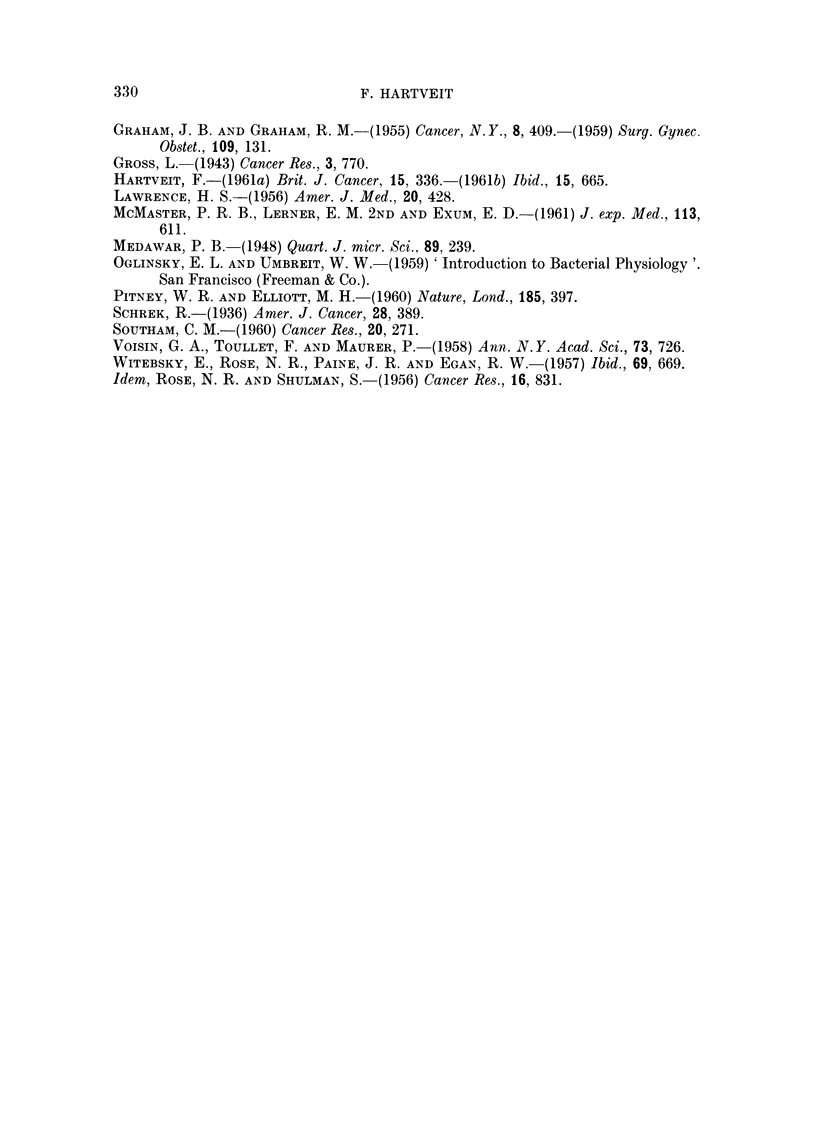# Evidence of Induced Immunity to Ehrlich's Ascites Carcinoma Brought About Through the Combination of Freund's Adjuvant with Living Tumour

**DOI:** 10.1038/bjc.1962.37

**Published:** 1962-06

**Authors:** F. Hartveit


### EVIDENCE OF INDUCED IMMUNITY TO EHRLICH'S ASCITES CARCINOMA BROUGHT ABOUT THROUGH THE COMBINATION OF FREUND'S ADJUVANT WITH LIVING TUMOUR

F. HARTVEIT

*From the University of Bergen, School of Medicine, the Gade Institute, Department of Pathology, Bergen, Norway*

Received for publication February 27, 1962

IN this experiment living Ehrlich ascites carcinoma was combined with Freund's adjuvant in an attempt to induce immunity to the tumour. This adjuvant has been shown to enhance antibody production and to produce a specific allergic type response to hetero-, homo-, and autotransplants (Freund, 1956 ; Witebsky, Rose, Paine and Egan, 1957 ; McMaster, Lerner and Exum, 1961). Both serum antibodies and delayed sensitivity have been reported following its use (Freund, 1956). Medawar (1948) has shown that delayed sensitivity is involved in the rejection of tissue transplants, and it is also involved in the rejection of tumour transplants (Lawrence, 1956).

It has been shown that a combination of dead tumour in Freund's adjuvant may result in the production of tumour antibodies (Fink, Smith and Rothlauf, 1955 ; Witebsky, Rose and Shulman, 1956 ; Graham and Graham, 1959 ; Finney, Byers and Wilson, 1960), but these are not easy to demonstrate. In the present experiment living tumour was used to avoid the possibility of denaturating the tumour protein, and subcutaneous injection was chosen as it is thought to be optimal for the development of delayed sensitivity (Lawrence, 1956). The blood content of the tumour ascites was used as a measure of the tumour immunity (Hartveit, 1961*b*).

### MATERIAL AND METHODS

*Mice.*—These were similar to the mice used in previous experiments (Hartveit, 1961*b*) and were selected in the same way. Eight groups of 15 males and 15 females were set up.

*Tumour.*—The Ehrlich ascites carcinoma used was taken from the 84th transplant generation of the tumour maintained at this Institute.

*Preparation of the adjuvant.*—Freund's method (1956) for the preparation of complete adjuvant was followed. Eucerin anhydrous was used as the emulsifying agent. The tubercle bacilli were of strain H37, Rv (kindly supplied by Professor Vogelsang, Gade Institute, Department of Microbiology). Four ml. of this adjuvant mixture were emulsified with 4 ml. of saline.

*Preparation of tumour inoculum.*—The tumour ascites from two male and two female mice that had been injected intraperitoneally with Ehrlich's ascites carcinoma 10 days previously, was pooled. The resulting fluid contained 2,110,000 tumour cells/mm$^3$ ; PCV 31 per cent, and 1 per cent packed erythrocytes. All the tumour cells were considered viable as none stained by Schrek's method (1936). Four ml. of this ascites were emulsified with 4 ml. of the adjuvant mixture. A

further quantity was diluted with saline to give the same tumour cell dose/unit volume as in the tumour-adjuvant mixture. The remaining fluid was used undiluted.

*Experimental procedure.*—The treatment given to the mice is summarized in Table I. The intraperitoneal tumour dose was 0·1 ml. The subcutaneous injections (0·05 ml.) were given on the back. When the injections had been completed only an occasional tumour cell in the remaining fluid was of doubtful viability.

TABLE I.—*The Treatment given to the Mice in the Different Groups. (EAC = Ehrlich's ascites carcioma. FA = Freund's adjuvant)*

| | Route of injection | |
|---|---|---|
| Group | Subcutaneous | Intraperitoneal |
| 1 . | nil | nil |
| 2 . | nil | EAC |
| 3 . | FA + EAC | nil |
| 4 . | FA + saline | nil |
| 5 . | EAC + saline | nil |
| 6 . | FA + EAC | EAC |
| 7 . | FA + saline | EAC |
| 8 . | EAC + saline | EAC |

Thereafter any subcutaneous growths were measured weekly. When a mouse died the survival time was recorded in days. If a subcutaneous growth was present this was measured. If the mouse had been given an intraperitoneal injection 1 ml. of the ascites was centrifuged and the PCV of the erythrocytes recorded (Hartveit, 1961*a*).

### RESULTS

*Survival time.*—Table II gives the mean survival time of the mice that received intraperitoneal tumour. The differences between the groups are not significant for the total or male series. The females in groups 6 and 7 died significantly earlier than those in group 8 ($0·05 > P > 0·02$ and $0·01 > P > 0·001$, respectively). The females in group 7 also died earlier than the males in the same group ($0·01 > P > 0·001$). The sex difference in the other 3 groups is not statistically significant.

All the mice in the remaining control groups were alive a week after the last mouse with intraperitoneal tumour died ; showing that the mice themselves and their surroundings were healthy, and that the subcutaneous treatment did not kill the mice within the time limit of the experiment. The survival times of these animals will be discussed in a later paper.

TABLE II.—*The Mean Survival Time (with SD) of the Mice in the Groups with Intraperitoneal Tumour. (30 mice in each group)*

| | Mean survival time (days) | | |
|---|---|---|---|
| Group | Total series | Male series | Female series |
| 2 . | 12·8 ± 2·944 | 13·86 ± 3·178 | 11·73 ± 2·220 |
| 6 . | 11·7 ± 3·435 | 12·8 ± 3·661 | 10·6 ± 2·846 |
| 7 . | 11·93 ± 2·497 | 13·26 ± 2·657 | 10·6 ± 1·342 |
| 8 . | 12·9 ± 1·817 | 13·06 ± 1·378 | 12·73 ± 2·168 |

*The blood content of the tumour ascites.*—This was measured in groups 2, 6, 7 and 8, and the mean percentages are shown in Table III. The greatest amount of blood was present in group 6. Groups 2 and 7 showed approximately the same amount for the total series, while group 8 showed least. The difference between groups 6 and 8 is significant for the total and female series (0·01 > P > 0·001 and 0·02 > P > 0·01, respectively). The sex difference was only significant in group 7 in which the males showed less blood than the females (0·001 > P). As a result of this the difference in blood content between the males in groups 6 and 7 becomes significant (0·05 > P > 0·02).

TABLE III.—*The Mean Blood Content of the Ascites (with SD) in the Mice with Intraperitoneal Tumour. (30 mice in each group)*

| | Mean blood content (per cent) | | |
|---|---|---|---|
| Group | Total series | Male series | Female series |
| 2 . | 5·1 ± 2·902 | 4·26 ± 2·755 | 5·93 ± 2·795 |
| 6 . | 6·931 ± 4·842 | 6·0 ± 4·957 | 7·8 ± 4·564 |
| 7 . | 5·0 ± 2·532 | 3·071 ± 1·594 | 6·8 ± 1·797 |
| 8 . | 3·86 ± 2·565 | 3·4 ± 2·184 | 4·3 ± 2·802 |

The results in male groups 6 and 7 are based on 14 mice as too little ascites was present in the remaining two.

*The correlation between the survival time and the blood content of the ascites (groups 2, 6, 7 and 8).*—Table IV shows that this negative correlation is highest in the control group 2 (0·001 > P), and next highest in group 6 (0·001 > P), the experimental group. It is statistically significant in all groups but group 7.

TABLE IV.—*Correlation Between Survival Time and Blood Content of the Ascites in Mice with Intraperitoneal Tumour. (30 mice in each group)*

| | Correlation coefficient ($r$) | | |
|---|---|---|---|
| Group | Total series | Male series | Female series |
| 2 . | −0·8383 | −0·8774 | −0·7700 |
| | (0·001 > $P$) | (0·01 > $P$ > 0·001) | (0·02 > $P$ > 0·01) |
| 6 . | −0·7905 | −0·7900 | −0·7844 |
| | (0·001 > $P$) | (0·02 > $P$ > 0·01) | (0·02 > $P$ > 0·01) |
| 7 . | −0·6195 | −0·2505 | −0·5542 |
| | (0·01 > $P$ > 0·001) | (0·4 > $P$ > 0·3) | (0·1 > $P$ > 0·5) |
| 8 . | −0·6653 | −0·7638 | −0·5981 |
| | (0·01 > $P$ > 0·001) | (0·02 > $P$ > 0·01) | (0·05 > $P$ > 0·02) |

Results based on 14 male mice in groups 6 and 7.

For the purposes of the following calculations the mean survival time of the animals in group 2 (12·8 days) was taken as the dividing line between those with a short (a) and those with a long (b) survival time following intraperitoneal injection of the tumour.

*The distribution of the survival time of the mice within the groups (2, 6, 7 and 8).*—Table V shows that the mice in groups 2 and 8 were more or less evenly distributed between groups a and b, while group 7 showed more, and group 6 even more, mice in group a. In groups 2 and 8 the sex difference in this distribution is not significant, while it is so in groups 6 and 7 ($0{\cdot}01 > P > 0{\cdot}001$) as many more females than males fall into group a. For the total series the difference in the percentage of mice in group a is significant between groups 6 and 8, and for the females between groups 6 and 8 ($0{\cdot}01 > P > 0{\cdot}001$) and 7 and 8 ($0{\cdot}05 > P > 0{\cdot}02$).

TABLE V.—*The Distribution of the Survival Time of the Mice with Intraperitoneal Tumour within the Groups, giving Number of Mice (per cent)*

| | Survival time | | | | | |
|---|---|---|---|---|---|---|
| | Short (a) | | | Long (b) | | |
| Group | Male | Female | Total | Male | Female | Total |
| 2 . | 5 | 10 | 15 (50) | 10 | 5 | 15 (50) |
| 6 . | 7 | 14 | 21 (72·4) | 7 | 1 | 8 (27·6) |
| 7 . | 5 | 13 | 18 (62·1) | 9 | 2 | 11 (37·9) |
| 8 . | 6 | 8 | 14 (46·7) | 9 | 7 | 16 (53·3) |

The results in male groups 6 and 7 are based on 14 instead of 15 mice as the ascites in the remaining two was not suitable for further investigation.

*The distribution of the mean blood content of the tumour ascites (per cent) according to the survival time within the groups* (2, 6, 7 *and* 8).—Table VI shows that in all cases there was more blood in group a than b. This difference was statistically significant in all groups. The sex difference within the groups was significant in group 7a ($0{\cdot}001 > P$), and in group 6b (but the latter contains only one female mouse). For the total series the difference between groups 6a and 8a is significant ($0{\cdot}01 > P > 0{\cdot}001$), for the male series that between groups 6a and 7a, and for the females those between groups 2b and 6b and 2b and 8b ($0{\cdot}05 > P > 0{\cdot}02$).

TABLE VI.—*The Distribution of the Mean Blood Content of the Tumour (per cent), with SD, according to the Survival Time within the Groups of Mice with Intraperitoneal Tumour.* (30 *mice in each group*)

| | Survival time | | | | | |
|---|---|---|---|---|---|---|
| | Short (a) | | | Long (b) | | |
| Group | Male | Female | Total | Male | Female | Total |
| 2 . | 6·8 ±3·763 | 6·9 ±2·468 | 6·86±3·891 | 3·0 ±1·673 | 4·0 ±2·408 | 3·3 ±2·037 |
| 6 . | 8·7 ±5·719 | 8·36±4·201 | 8·47±4·659 | 3·29±1·375 | 0·0 ±0·0 | 2·9 ±1·458 |
| 7 . | 3·4 ±1·908 | 7·23±1·480 | 6·17±4·078 | 2·89±1·485 | 4·0 ±1·0 | 3·09±1·475 |
| 8 . | 4·0 ±1·965 | 5·62±2·287 | 5·0 ±2·3 | 2·89±5·509 | 2·86±2·641 | 2·88±2·367 |

The results in male groups 6 and 7 are based on 14 mice as too little ascites was present in the remaining two.

*The subcutaneous injection site.*—This was examined in all appropriate groups on the 7th day after injection. The findings are shown in Table VII. The three mice in group 6 that had died by this time showed thickening under the skin at the injection site, but no palpable masses. The differences in mean tumour diameter between groups 3, 5 and 8 are not statistically significant. Thus the addition of adjuvant to the tumour has not affected its vitality. The difference between groups 3 and 6 (Fig. 1), is significant ($0{\cdot}001 > P$). The sex difference is only significant in group 5 in which the tumours in the males were larger than those in the females ($0{\cdot}05 > P > 0{\cdot}02$).

FIG. 1.—Greatest subcutaneous tumour diameter 7 days after injection in group 3 (hatched columns) and group 6 (dark columns). Values for individual mice in each group given alternately. Note: The tumours were measured at autopsy in 3 mice in group 6.

TABLE VII.—*The Average Greatest Diameter (mm) of the Subcutaneous Tumours (with SD) at 7 Days*

| Group | Tumour diameter (mm.) | SD (mm.) |
|---|---|---|
| 3 | 9·73 | 2·112 |
| 5 | 9·166 | 3·976 |
| 6 | 1·4 | 1·698 |
| 8 | 7·13 | 4·249 |

All the mice that had been given adjuvant without tumour subcutaneously (groups 4 and 7) showed slight thickening at the injection site on the 7th day. At 14 days in group 4, and at 14 days or at autopsy in group 7, the site was difficult to locate in the males while all the females showed a distinct freely movable swelling (approximately 0·5 cm. in diameter) in the subcutaneous tissues. Ulceration did not occur.

### DISCUSSION

The majority of attempts to detect serum antibody to tumour tissue have failed (Barrett, 1958) and some of the serum antibodies that have been reported (Southam, 1960) may have been due to genetic differences between host and tumour and not to the fact that the tissue was tumour tissue. For there to be a genetic difference between a host and its own tumour a mutation must have occurred. The mutation, in altering the function of the mutant gene (Oglinsky and Umbreit, 1959), may result in a change in the protein synthesised. This protein may then be recognized as " non-self " : a change in a surface protein producing serum antibody, one deep in the cell giving delayed sensitivity. But if the mutation results in a change in the rate of protein synthesis leaving the structure unchanged (Brinkhaus and Graham, 1954 ; Pitney and Elliott, 1960), no recognition can be expected. This, or a change in an intracellular protein, could explain the almost universal failure to detect serum antibody to autologous tumours. In the former case any enhancing action shown by Freund's adjuvant could be due to the mobilization of immunologically active cells (Burnet, 1959). In the latter some factor in the adjuvant may combine with the " self " protein, the combination being recognized immunologically (Freund, 1956). Such haptene formation is thought to occur in the autoimmune diseases (Favour, 1956 ; Voisin, Toullet and Maurer, 1958 ; Asherson and Broberger, 1961).

In reactions involving normal autologous tissues the second mode of action must be postulated as the possibility of genetic difference does not exist. While reaction against the protein-haptene complex could be expected, reaction against the original protein has, in fact, been demonstrated (McMaster, Lerner and Exum, 1961). Thus, it is not necessary to postulate a genetic difference between host and tumour to explain the rationale of using a combination of autologous tumour and Freund's adjuvant in an attempt to produce changes in a distant transplant of the tumour.

While the tumour used in this experiment is a homograft it is known that some of the mice used will accept it as an autograft, the blood content of the tumour ascites being a measure of their natural immunity (Hartveit, 1961*b*). The present experiment was designed to see if subcutaneous treatment with Freund's adjuvant combined with living tumour would increase the immunological response of the mice to the tumour and, if this were so, whether the increase was in natural or acquired resistance. To show this the distribution of the mice within the groups was investigated. The mean survival time of the animals in the control group 2 was taken as the dividing line between those with a short (a) and those with a long survival time (b) ; the mice in group a being those resisting the tumour, group b accepting it. If the treatment resulted in a greater number of mice in group a than could be expected on the basis of the findings in the controls—it would then have been shown that immunity had been induced in some of the mice that would normally have accepted the tumour without response.

The results show that the mice in group 8 had the longest survival time, the least blood in the tumour ascites and the highest number of mice in group b. Thus these mice show most evidence of accepting the tumour homograft, even more than the untreated control group 2. This may be an example of an XYZ effect (Casey, Hatherway and Casey, 1956 ; Goldie, Walker, Kelley and Gaines, 1956) brought about through a decrease in the immunological response of the host. This is in keeping with clinical experience of malignant growths (Graham and Graham, 1955). As the mice in this group show least evidence of natural immunity they are, in fact, more suitable than group 2 as a control for the experimental group 6.

The following differences between group 6 and 8 are statistically significant. The females in group 6 died earlier than those in 8. There was more blood in the ascites in group 6 than in group 8. Group 6 showed a higher percentage of mice in group a than did group 8, and there was a higher blood content in the ascites in 6a than 8a.

These results consistently indicate that the subcutaneous tumour-adjuvant mixture increased the immunological response of the mice to the intraperitoneal tumour. The effect is particularly marked in the females in group 6 only one of which remained in group b, i.e. was unaffected by the adjuvant-antigen mixture. The increase in the tumour blood content shows that the immunity of the mice which already possess some natural resistance has been strengthened. The increase in the number of mice in group a shows that immunity has been induced in some mice that previously lacked resistance. As it has proved possible to measure both natural and acquired immunity through the blood content of the tumour it is probable that the same mechanism is at work in both cases.

As the mice in group 7 received adjuvant alone subcutaneously the decrease in survival time and increase in tumour blood content in the female mice must be regarded as non-specific. Non-specific allergic responses have been reported with normal tissues following the injection of adjuvant alone (Voisin, Toullet and Maurer, 1958), but a marked sex difference has not been described previously. This sex difference is further reflected in the reaction at the subcutaneous injection site, which was marked in the females and absent in the males. As the findings in group 4 were similar the reaction is not dependent on the presence of intraperitoneal tumour. It is often easier to produce specific tumour immunity in female mice (Gross, 1943) as is shown in group 6. It appears that non-specific immunity also is easier to produce in females.

This non-specific immunity must differ in some way from that produced by adjuvant combined with antigen as the normal relationship between the survival time and the tumour blood content, that holds in group 6, has been upset in group 7. It may be that the mode of action of adjuvant alone differs from that of an adjuvant-antigen mixture. While the adjuvant alone will be able to mobilize immunologically active cells, haptene formation will not be possible. This strengthens the idea that haptene formation is concerned in the adjuvant-antigen response to tumour tissue.

It was also found that in mice with intraperitoneal tumour, the tumour in the adjuvant-antigen mixture failed to grow as quickly as in the mice without a further source of tumour (Table VII and Fig. 1). This is contrary to what would be expected on the basis of an XYZ effect, and is difficult to explain. It may be that the intraperitoneal tumour stimulates the sensitivity reaction by neutralizing its products.

### SUMMARY

Freund's adjuvant given subcutaneously in combination with living Ehrlich ascites carcinoma was found to increase the immune response of mice to the intraperitoneal injection of the same tumour. Both natural and acquired immunity appear to have been affected. An increase in the immune response was also found in female mice following Freund's adjuvant alone. Evidence is presented that the mechanism of this non-specific reaction differed from that of the specific reaction. The combination of adjuvant and living tumour had an inhibitory effect on the growth of the tumour in the adjuvant mixture when intraperitoneal tumour was also present.

### REFERENCES

Asherson, G. L. and Broberger, O.—(1961) *Brit. med. J.*, i, 1429.
Barrett, M. K.—(1958) *J. chron. Dis., St. Louis*, **8**, 136.
Brinkhaus, K. M. and Graham, J. B.—(1954) *Blood*, **9**, 255.
Burnet, F. M.—(1959) 'The Clonal Selection Theory of Acquired Immunity'. Cambridge (The University Press), p. 149.
Casey, A. E., Hatherway, E. A. and Casey, J. G.—(1956) *Cancer Res.*, **16**, 324.
Favour, C. B.—(1956) *Ann. N.Y. Acad. Sci.*, **64**, 842.
Fink, M. A., Smith, P. and Rothlauf, M. V.—(1955) *Proc. Soc. exp. Biol., N.Y.*, **90**, 590.
Finney, J. W., Byers, E. H. and Wilson, R. H.—(1960) *Cancer Res.*, **20**, 351.
Freund, J.—(1956) *Advanc. tuberc. Res.*, **7**, 130.
Goldie, H., Walker, M., Kelley, L. and Gaines, J.—(1956) *Cancer Res.*, **16**, 553.

Graham, J. B. and Graham, R. M.—(1955) *Cancer, N.Y.*, **8**, 409.—(1959) *Surg. Gynec. Obstet.*, **109**, 131.
Gross, L.—(1943) *Cancer Res.*, **3**, 770.
Hartveit, F.—(1961*a*) *Brit. J. Cancer*, **15**, 336.—(1961*b*) *Ibid.*, **15**, 665.
Lawrence, H. S.—(1956) *Amer. J. Med.*, **20**, 428.
McMaster, P. R. B., Lerner, E. M. 2nd and Exum, E. D.—(1961) *J. exp. Med.*, **113**, 611.
Medawar, P. B.—(1948) *Quart. J. micr. Sci.*, **89**, 239.
Oglinsky, E. L. and Umbreit, W. W.—(1959) ‘ Introduction to Bacterial Physiology ’. San Francisco (Freeman & Co.).
Pitney, W. R. and Elliott, M. H.—(1960) *Nature, Lond.*, **185**, 397.
Schrek, R.—(1936) *Amer. J. Cancer*, **28**, 389.
Southam, C. M.—(1960) *Cancer Res.*, **20**, 271.
Voisin, G. A., Toullet, F. and Maurer, P.—(1958) *Ann. N.Y. Acad. Sci.*, **73**, 726.
Witebsky, E., Rose, N. R., Paine, J. R. and Egan, R. W.—(1957) *Ibid.*, **69**, 669.
*Idem*, Rose, N. R. and Shulman, S.—(1956) *Cancer Res.*, **16**, 831.